# Functional metabolomics: unlocking the role of small molecular metabolites

**DOI:** 10.3389/fmolb.2025.1542100

**Published:** 2025-07-09

**Authors:** Hetao Chen, Jiao Kong, Peipei Du, Qian Wang, Tao Jiang, Xixi Hou, Tingting Feng, Jiajia Duan, Chuanxin Liu

**Affiliations:** ^1^ Luoyang Key Laboratory of Clinical Multiomics and Translational Medicine, Henan Key Laboratory of Rare Diseases, Endocrinology and Metabolism Center, The First Affiliated Hospital, and College of Clinical Medicine of Henan University of Science and Technology, Luoyang, China; ^2^ Department of Clinical Laboratory, The First Affiliated Hospital, College of Clinical Medicine of Henan University of Science and Technology, Luoyang, China; ^3^ Institute of Drug Metabolism and Pharmaceutical Analysis, College of Pharmaceutical Sciences, Zhejiang University, Hangzhou, China; ^4^ School of Chinese Materia Medica, Beijing University of Chinese Medicine, Beijing, China; ^5^ Department of Pharmacy, The First Affiliated Hospital, and College of Clinical Medicine of Henan University of Science and Technology, Luoyang, China

**Keywords:** functional metabolomics, metabolic diseases, chemical biology, molecular biology, liquid chromatography-mass spectrometry

## Abstract

Metabolomics is an expanding field dedicated to elucidating metabolic disorders by analyzing endogenous small molecules in biological samples. With the rapid advancement of metabolomics, researchers are investigating the influence of metabolites on metabolic phenotypes. The emergence of functional metabolomics provides a methodological framework to address this issue. This approach focuses on the biological functions of metabolites and their corresponding enzymes while validating the potential mechanisms of differential metabolites through *in vivo* and *in vitro* experiments. Despite numerous research findings, a systematic compilation of case studies remains absent. Therefore, this review systematically summarizes and evaluates functional metabolomics, covering its historical development, current state, and future directions, with the aim of fostering its advancement and offering solutions for further research.

## 1 Introduction

Small-molecule metabolites, as substrates, precursors, and metabolic byproducts, play a crucial role in physiological processes. In 1997, Stephen Oliver et al. introduced the concept of the metabolome by evaluating yeast gene functions through metabolite analysis (Oliverp et al., 1998). In 1999, Nicholson et al. from Imperial College London defined metabonomics as “the quantitative measurement of the dynamic metabolic response of living systems to stimuli or genetic modifications” (Nicholson et al., 1999). In 2000, Fiehn et al. defined metabolomics as the static measurement of all low-molecular-weight metabolites (M < 1,500 Da) in an organism at a specific time under defined conditions (Fiehn et al., 2000). Presently, metabolomics focuses on analyzing small molecules across various biological matrices (cells, tissues, fluids, and plant extracts), forming an integral component of systems biology. Over the past 2 decades, metabolomics has significantly advanced disease diagnosis, plant genomics, toxicology, and microbial research. For instance, in pancreatic cancer, carbohydrate antigen (CA19-9) serves as a primary diagnostic marker. Mayerle et al. (Mayerle et al., 2018) identified the characteristics of CA19-9 alongside nine metabolites, enhancing diagnostic accuracy. Matsumoto et al. (Matsumoto and Kuhara, 1996) developed an optimized UP-GC-MS method for neonatal organic aciduria screening, facilitating the simultaneous analysis of over 200 abnormal metabolites. This method was introduced in major Chinese cities in 2000 (Jiang et al., 2015). Luo et al. (Luo et al., 2018) identified two early liver cancer markers: phenylpropionyl tryptophan and hepatocholic acid, with the latter receiving clinical approval for bile acid-related diseases. Metabolomics enables early-stage “liquid biopsy” diagnosis of asymptomatic diseases and provides metabolic profiles for traditional Chinese medicine syndromes (Li et al., 2024; Nicholson et al., 2005; Johnson et al., 2016). However, challenges persist, including the lack of standardized general databases (Fiehn, 2002).

Traditionally, metabolomics, also referred to as “phenotypic metabolomics,” “discovery metabolomics,” or “classical metabolomics,” comprises four essential steps: sample preparation, data collection, data processing, and biological interpretation. Oliver Fiehn classified metabolic analysis into four hierarchical levels: metabolite target analysis, metabolic profiling analysis, metabolomics, and metabolic fingerprint analysis (Fiehn, 2002). Over time, metabolomics has evolved into two principal categories: non-targeted and targeted metabolomics. Non-targeted metabolomics employs high-resolution mass spectrometry to obtain comprehensive metabolite information; however, it is characterized by data complexity, limited reproducibility, and a narrow linear range. Conversely, targeted metabolomics focuses on specific small molecules and pathways, offering higher accuracy at the expense of reduced coverage. To overcome these limitations, in 2012, Xu Guo-Wang’s research team introduced pseudotargeted metabolomics, which integrates the strengths of both approaches by employing a quantitative ion selection algorithm to quantify all detected metabolites (Xuan et al., 2018). This method was later adapted for liquid chromatography-mass spectrometry applications (Chen et al., 2013).

**FIGURE 1:**
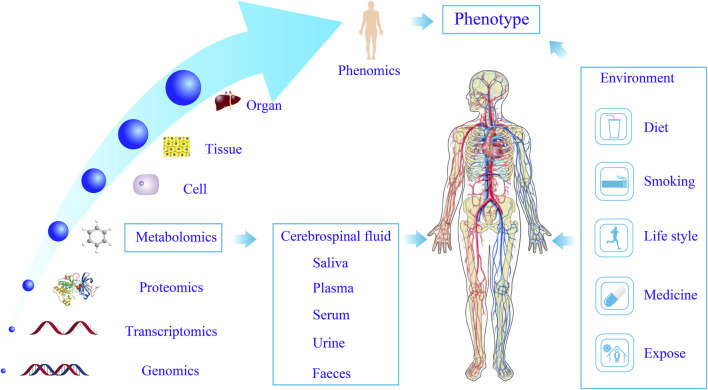
Systems biology characterizes the objective phenotype of the human body. Metabolomics enables dynamic biological system profiling through multi-omics integration, advancing disease diagnostics, drug discovery, and the elucidation of disease mechanisms.

Functional metabolomics, a novel extension of traditional metabolomics, emphasizes the functional roles of metabolites and their associated enzymes. It complements phenotypic metabolomics by prioritizing *in vivo* and *in vitro* experimental validation of metabolite functions (Yan and Xu, 2018). By integrating genetics, isotope tracing, and other advanced techniques, Zeneng et al. (Wang et al., 2019; Wang et al., 2011) and Robert et al. (Koeth et al., 2013) identified key precursor metabolites of trimethylamine N-oxide (TMAO) implicated in diabetes and cardiovascular diseases. Functional metabolomics facilitates a deeper exploration of metabolite functions and their interactions with genes and proteins, positioning itself as a key trend in contemporary metabolomics research. While traditional metabolomics enables the rapid screening of metabolites and metabolic pathways, functional metabolomics validates the biological significance of identified metabolites. Furthermore, variations in small molecules offer a more precise reflection of physiological states compared to genomics, proteomics, and transcriptomics. Functional metabolomics allows dynamic monitoring of biological systems, and through multi-omics integration in systems biology, it provides a holistic understanding of human structure and function. This approach elucidates the interplay between endogenous small molecules and macromolecules (Q et al., 2020), facilitates the identification of early diagnostic biomarkers, enables disease classification, uncovers novel drug targets, evaluates therapeutic efficacy, and elucidates disease mechanisms (Liu et al., 2020) (Figure 1).

In conclusion, this review aims to provide a comprehensive analysis of the evolution, current state, and future prospects of functional metabolomics. It highlights the potential of this field in addressing the limitations of traditional metabolomics and advancing our understanding of metabolite functions and their corresponding enzymes. Additionally, this review explores the applications of functional metabolomics in disease diagnosis and treatment, as well as its integration with other omics technologies to achieve a more profound understanding of complex biological systems.

## 2 Research strategies in functional metabolomics

**FIGURE 2:**
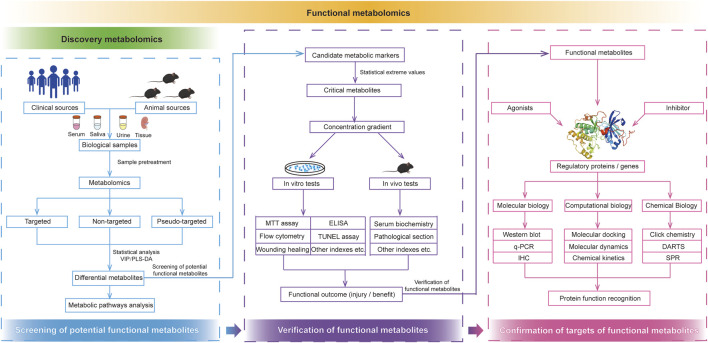
Research strategies of functional metabolomics. Functional metabolomics mainly divided into the screening of potential functional metabolites, verification of functional metabolites, and confirmation of targets of functional metabolites.

The continuous advancement of analytical methods with high throughput, sensitivity, quantitative accuracy, and reproducibility is a fundamental objective of classical metabolomics. Unlike classical metabolomics, functional metabolomics places greater emphasis on elucidating the biological functions of endogenous small-molecule metabolites within the body. The research strategies employed in functional metabolomics are illustrated in Figure 2 and can be broadly categorized into three key stages: screening of potential functional metabolites, validation of functional metabolites, and identification of regulatory targets.

Functional metabolomics is guided by classical metabolomics, which screens differential small-molecule metabolites most relevant to phenotypic variations and provides candidates for subsequent biological function analysis. However, the number of potential markers identified ranges from a few to several dozen, necessitating a strategic selection of key small-molecule metabolites from this complex dataset. Previous studies have demonstrated that the identification of crucial small-molecule metabolites primarily relies on the following strategies (Oliverp et al., 1998): Extreme change multiple: If the concentration or abundance of one or more endogenous components undergoes a significant alteration before and after an intervention, it is highly likely to be a key metabolite with potential biological functions. Under normal physiological conditions, the levels of endogenous metabolites maintain a dynamic equilibrium within a specific range. Once small molecules exhibiting extreme changes are identified through discovery metabolomics, they warrant further investigation as potential functional targets. (Nicholson et al., 1999). Abnormal statistical effect quantity: In functional metabolomics, advanced statistical methods such as partial least squares discriminant analysis (PLS-DA) and variable importance in projection (VIP) scores play a crucial role in identifying biologically significant metabolites. PLS-DA facilitates data dimensionality reduction, enabling the differentiation of sample groups based on metabolite profiles (Ji-ye, 2010). Meanwhile, VIP scores quantify the contribution of individual metabolites to this classification, with a threshold of >1.0 commonly employed to prioritize candidates for further validation (Hayashi and Iwata, 2015). Unlike conventional p-values, VIP scores address multicollinearity in metabolomics data, making them indispensable for detecting subtle metabolic changes associated with specific phenotypes. Statistical rigor is further enhanced through complementary approaches, including: unsupervised dimensionality reduction via principal component analysis (PCA) for detecting global clustering patterns; rigorous differential abundance testing using Welch’s t-test (for normally distributed data) or Mann-Whitney U test (for non-parametric data), with false discovery rate (FDR) correction to control Type I errors in high-dimensional datasets; comprehensive visualization strategies such as box plot, volcano plot and hierarchically clustered heatmaps, and receiver operating characteristic (ROC) curves for assessing key metabolites performance; and systematic normalization techniques, including the use of internal standards, probabilistic quotient normalization, or total ion current alignment to correct technical variations. For instance, Wang et al. (Wang et al., 2021) utilized one-way analysis of variance (ANOVA) to identify novel molecular subgroups and assessed the diagnostic performance of hub ATGs in 40 clinical samples and human primary endometrial stromal cells (ESCs) using ROC curve analysis. These statistical tools bridge the gap between discovery and validation, facilitating targeted studies of metabolites such as TMAO in cardiovascular diseases (Spence, 2023). This integration ensures that statistical findings translate into actionable biological insights, aligning with the overarching goal of elucidating metabolite-driven pathways (Fiehn et al., 2000). Multi-Omics correlation analysis: In the post-genomic era, systems biology, which integrates multiple omics layers, has significantly enhanced the screening of key small-molecule metabolites. For a given phenotype, cross-validation among different omics datasets offers novel opportunities for identifying functionally relevant metabolites. By integrating transcriptomic, proteomic (including post-translational modifications), and microbiome data, correlation analyses can systematically associate key mRNAs, proteins, and microbial abundances with metabolite fluctuations. These multi-omics approaches provide robust frameworks for identifying functional metabolites, although the selection of appropriate methodologies must be tailored to specific research contexts. For instance, Zhao et al. (Zhao et al., 2025) integrated fecal metabolomics, gut microbiome profiling, and brain transcriptomics to identify microbial-derived bile acids (e.g., deoxycholic acid) that modulate neuronal inflammation via the AGEs-RAGE axis in Alzheimer’s disease, thereby establishing a mechanistic link between gut metabolites and cognitive decline. Similarly, Hensley et al. (Hensley et al., 2016) mapped metabolic heterogeneity in lung tumors by correlating single-cell RNA-seq data with LC-MS-based metabolomics, revealing compartment-specific accumulation of lactate and succinate driven by HIF-1α transcriptional activity. These studies exemplify how multi-omics integration elucidates causal relationships across biological layers.

Following the identification of potential functional small molecules, it is imperative to conduct *in vivo* and *in vitro* experiments to systematically evaluate their biological functions. Typically, a series of concentration gradients for target metabolites is established, and these metabolites are administered to model organisms. The phenotypic indicators associated with the animal model are subsequently assessed to determine the protective or detrimental effects of small-molecule metabolites on specific target organs or phenotypic outcomes. Concurrently, *in vitro* experiments employing cellular assays, including MTT assays, enzyme-linked immunosorbent assays (ELISA), fluorescence microscopy colocalization, immunohistochemistry, and cell migration/scratch assays, are conducted to evaluate the impact of key metabolites on effector cells. For instance, Saeedi et al. (Saeedi et al., 2019a) employed MTT assays to verify the non-cytotoxicity of novel anti-diabetic compounds in HepG2 cells, whereas Dwivedi et al. (Dwivedi and Jena, 2020) utilized ELISA to quantify the suppression of IL-6 and TGF-β by glibenclamide in NAFLD models, thereby connecting metabolite effects to inflammatory pathways. These experiments help determine whether *in vivo* and *in vitro* findings are consistent and whether the observed effects are synergistic.

Based on the biological outcomes obtained, molecular biological techniques such as quantitative polymerase chain reaction (qPCR), immunoblotting, molecular docking, molecular dynamics simulations, and surface plasmon resonance (SPR) are utilized to further investigate the signaling pathways through which key metabolites exert protective or detrimental effects. These analyses continue until the molecular mechanisms underlying their biological functions are elucidated.

Once the biological functions of small-molecule metabolites have been established, the next crucial question pertains to their target proteins and their potential biological effects. Are these proteins viable therapeutic targets? Subsequent research focuses on the analysis and evaluation of key target proteins. By administering corresponding agonists or inhibitors to key target proteins, functional evaluations can be performed using computational biology, chemical biology, and molecular biology techniques, including gene knockdown, overexpression, interference, and inhibition assays. These approaches enable systematic assessment of the target protein’s function upon stimulation, ultimately allowing the determination of its biological activity and relevance to metabolite-mediated effects.

Previous studies have demonstrated the effectiveness of untargeted metabolomics in linking gut microbiota-derived metabolites, such as arachidonic acid, to the progression of Alzheimer’s disease (Zhao et al., 2025). This association was validated using mediation models and computational tools, with false discovery rate (FDR) correction for multiple comparisons and principal component analysis (PCA) for dimensionality reduction. These methodologies align with established strategies, including extreme variation magnitude and statistical significance in multivariate analyses (e.g., VIP scores). Similarly, targeted metabolomics and lipidomics have been employed to quantify serum metabolites, such as triglycerides, in chronic metabolic diseases, with validation through logistic regression and liquid chromatography-mass spectrometry (LC-MS) analysis (Zhou et al., 2024). Both studies underscore the importance of interdisciplinary validation methods, including statistical modeling and molecular profiling, in the identification of key functional metabolites.

Following the identification of candidate metabolites, systematic *in vivo* and *in vitro* experiments—including dose-response animal models, cell viability assays (MTT), ELISA, molecular biology techniques (qPCR, western blotting, and immunohistochemistry), computational biology methods (molecular docking, molecular dynamics, and chemical kinetics), and chemical biology approaches (click chemistry, drug affinity responsive target stability [DARTS], and SPR)—are essential for confirming biological effects and elucidating mechanistic pathways. The functional study of key metabolic small molecules constitutes a comprehensive and systematic investigation encompassing screening, validation, and regulatory analysis. It is essential to strategically integrate various experimental methodologies, leveraging their respective strengths while mitigating their limitations. Moreover, cross-disciplinary collaboration and integration of multiple fields are indispensable for the comprehensive and efficient identification of biologically active small-molecule metabolites.

## 3 Data acquisition and biological platform of functional metabolomics

In contrast to traditional metabolomics, functional metabolomics includes an additional step to determine the biological effects and molecular mechanisms of metabolites involved in metabolic pathways. Consequently, functional metabolomics not only necessitates the establishment of the detection platform and comprehensive database required by traditional metabolomics but also requires a verification platform for functional and mechanistic studies in cellular, animal, and clinical settings.

### 3.1 Data acquisition platform

Functional metabolomics employs the same detection techniques as traditional metabolomics, which are primarily categorized into three types: gas chromatography-mass spectrometry (GC-MS), LC-MS, and nuclear magnetic resonance (NMR). A summary of these three detection techniques is provided in Table 1. In addition to these methodologies, Fourier-transform mass spectrometry, capillary electrophoresis-mass spectrometry, and inductively coupled plasma mass spectrometry, among others, can also be utilized to analyze complex metabolites.

**TABLE 1:** Three major detection technologies of metabolomics.

Technologies	Commonly used instruments	Applicable substances	Advantages	Disadvantages	Optimal metabolite classes	Practical applications
GC/MS	GC/Q-MS GC/TOF-MS GC/IT-MS-MS	Easy to gasify, stable, not easy to decompose, not easy to react	Resolution, sensitivity, and reproducibility are good; the database is relatively complete	Complicated sample preparation and limited options; limited sample options	Volatiles (SCFAs), organic acids, sugars	GC/MS reveals metabolic perturbations in medullary thyroid carcinoma (Jajin et al., 2022)
LC/MS	LC/Q-MS UPLC/Q-MS LC/Q-TRAP-MS-MS UPLC/Q-TOF-MS-MS	Extensive	Good resolution, sensitivity and repeatability; many types of samples are available	Vulnerable to matrix effects	Lipids, peptides, polar metabolites	Global Metabolomics Using LC-MS for Clinical Applications (Skogvold et al., 2025)
NMR	LC/NMR LC/SPE-CRYONMR-MS	Not suitable for low-abundance metabolites	Simple pretreatment; qualitatively accurate; *in-situ* non-destructive testing	Low sensitivity; not suitable for quantification	small molecule metabolites, peptides	NMR Spectroscopy for Metabolomics in the Living System (Peng et al., 2024b)

GC-MS typically employs mass spectrometers such as triple quadrupole and ion trap instruments. The triple quadrupole mass spectrometer is characterized by high sensitivity and selectivity, with unit mass resolution, enabling multi-reaction monitoring (MRM) mode (Rontani, 2022). MRM specifically monitors selected precursor and product ions, effectively minimizing matrix interference and improving the precision of quantitative analysis. In contrast, the ion trap mass spectrometer exhibits high sensitivity and supports multi-stage mass spectrometry (MS^n^) analysis, making it ideal for structural elucidation of trace metabolites in complex matrices. In GC-MS analysis, parameters such as carrier gas flow rate, column temperature programming, and ionization energy significantly influence separation efficiency and detection performance (Hecht et al., 2016). For example, electron ionization (EI), a widely adopted ionization technique in GC-MS, operates at an ionization energy of 70 eV, producing stable ion fragments that facilitate accurate metabolite identification (Tsikas, 2024). GC-MS is well-suited for analyzing volatile or derivatized metabolites, such as short-chain fatty acids and sugars, offering high resolution and sensitivity. The availability of standardized spectral libraries, such as NIST, facilitates efficient metabolite identification (Fiehn et al., 2000). However, GC-MS requires time-consuming derivatization steps (e.g., silanization, methylation), which may introduce variability. It is not ideal for analyzing macromolecules or thermally unstable compounds. GC-MS is commonly employed for targeted analysis of energy metabolism intermediates, such as tricarboxylic acid (TCA) cycle metabolites (Calderón-Santiago et al., 2013). For instance, GC-MS combined with isotope tracing techniques (e.g., 13C-glutamine) enables detailed analysis of metabolic fluxes, providing insights into metabolic adaptations within the tumor microenvironment (Hensley et al., 2016).

LC-MS technology commonly utilizes Orbitrap, triple quadrupole, and ion mobility mass spectrometers (Pitt, 2009). Orbitrap mass spectrometers are distinguished by their exceptional resolution and accurate mass measurement capabilities, achieving resolutions exceeding 100,000. This feature provides substantial advantages for the precise identification and quantification of metabolites. The automatic gain control (AGC) function dynamically adjusts the ion injection time to ensure optimal signal intensity and resolution. Triple quadrupole mass spectrometers are widely employed in LC-MS for quantitative analysis through MRM (Kuzyk et al., 2013). By selecting specific precursor-product ion pairs and optimizing collision energy, they achieve highly sensitive and selective detection. Ion mobility mass spectrometers introduce an additional dimension of separation based on ion drift time, thereby enhancing the separation efficiency of complex samples and improving the accuracy of metabolite identification. In LC-MS analysis, factors such as the composition and gradient elution program of the mobile phase, the type and specifications of the chromatographic column, and electrospray ionization (ESI) conditions (e.g., spray voltage, sheath gas flow rate, auxiliary gas flow rate, etc.) significantly influence the separation and detection performance of metabolites (Timms and Cutillas, 2010). LC-MS integrates the high separation efficiency of liquid chromatography with the high sensitivity and selectivity of mass spectrometry, making it particularly suitable for analyzing polar macromolecules, thermally labile compounds, and complex biological matrices such as plasma and urine (Fiehn, 2001). Its non-targeted metabolomics mode allows for comprehensive coverage of a wide range of metabolites, including lipids, amino acids, and organic acids (Souza and Patti, 2021). High-resolution mass spectrometry (HRMS) provides accurate mass measurements, facilitating metabolite annotation and structural elucidation (Dunn et al., 2011). However, LC-MS is susceptible to matrix effects, such as ion suppression, necessitating sophisticated sample preparation techniques like solid-phase extraction. LC-MS is widely used in functional validation studies, particularly for dynamic metabolic flux analysis. For example, when combined with stable isotope labeling, LC-MS can track real-time intracellular metabolite transformations, revealing mechanisms of metabolic reprogramming, such as the Warburg effect observed in cancer cells (Reyes-Oliveras et al., 2024). Over the past 2 decades, LC-MS technology has undergone continuous advancements and has become a widely adopted tool in metabolomics research, showcasing distinct advantages over GC-MS. LC-MS exhibits extensive coverage, enabling direct analysis of polar, non-polar, thermally labile, and large molecular metabolites without derivatization, with a molecular weight range spanning 50–2000 Da (Dunn et al., 2011). Furthermore, LC-MS demonstrates high sensitivity and throughput. Modern high-resolution mass spectrometers, such as Q-TOF and Orbitrap, are capable of detecting low-abundance metabolites at the fg level. When coupled with ultra-high-performance liquid chromatography (UHPLC), these systems can achieve high-throughput analysis of 5–10 samples per minute (Zuo et al., 2021). Additionally, LC-MS possesses a wide dynamic range, with a linear response spanning four to six orders of magnitude, making it well-suited for analyzing biological samples with substantial concentration variations (Patti et al., 2012).

NMR offers non-destructive structural information on metabolites with high reproducibility and suitability for absolute quantification. It excels at distinguishing isomers, such as the α/β forms of glucose, outperforming mass spectrometry in this regard (Beckonert et al., 2010). However, its lower sensitivity (in the micromolar range) limits its ability to detect low-abundance metabolites. Data acquisition times are longer, making it less suitable for rapid quantification. NMR has unique advantages in dynamic metabolic tracking. For example, real-time monitoring of lactate and alanine dynamics in liver perfusion models can reveal time-dependent regulation of metabolic pathways (Peng et al., 2024a).

Overall, LC-MS is optimal for non-targeted metabolomics and dynamic flux analysis, GC-MS excels in targeted analysis of volatile metabolites, and NMR is indispensable for structural analysis and dynamic tracking. In functional metabolomics, integrating multiple techniques (e.g., LC-MS/NMR) leverages their complementary strengths to provide a comprehensive understanding of metabolite biological functions (Patti et al., 2012). Future research should focus on developing standardized workflows to enhance data comparability and leveraging AI-driven metabolic network modeling to accelerate the translation from mechanism discovery to clinical applications.

### 3.2 Function verification platform

#### 3.2.1 Cell biology platform

Cells constitute the fundamental units of biological activity. By observing and analyzing cellular responses, the effects of metabolite alterations on cell morphology, proliferation, migration, apoptosis, and metabolic activity can be elucidated. Ning et al. (Ning et al., 2024) investigated the role of melatonin in human retinal microvascular endothelial cell function *in vitro* using EdU incorporation assays, scratch assays, transwell assays, and tube formation tests. Similarly, Saeedi et al. (Saeedi et al., 2019b) employed MTT assays to confirm the non-cytotoxic nature of newly synthesized anti-diabetic compounds, demonstrating their potential as novel hypoglycemic agents. To elucidate the molecular mechanisms underlying the use of metformin in multiple myeloma treatment, Wang et al. (Wang et al., 2018) utilized CCK-8 assays to assess cell viability and flow cytometry to evaluate cell cycle distribution and apoptosis. Through these methods, they established that metformin inhibits myeloma cell proliferation by inducing autophagy and cell cycle arrest.

Recent studies have delineated multipotent cell populations and explored the relationship between aging, obesity, and fracture risk using flow cytometry and other technologies (Ambrosi et al., 2017). Dorrell et al. (Dorrell et al., 2016) identified four subtypes of human pancreatic islet β-cells using novel markers detected via flow cytometry. In 2016, the “Human Cell Atlas” (HCA) project was initiated (Regev et al., 2017) to define all human cell types based on molecular characteristics, associating gene expression and other information with classical cellular descriptions, such as morphology and spatial localization. This initiative aims to provide a foundational understanding of how cells contribute to complex organismal structures. A deeper comprehension of cellular characteristics will facilitate an improved understanding of molecular mechanisms at the cellular level.

#### 3.2.2 Animal model platform

Appropriate animal models serve as a cornerstone for mechanistic investigations. Taking diabetes as an example, rats, mice, zebrafish, and beagles can be selected based on specific research objectives. Spontaneous models of diabetes are challenging to obtain; therefore, researchers commonly induce diabetes using chemical agents, dietary interventions, or a combination of both. Additionally, surgical procedures or gene knockout techniques can be employed. In traditional Chinese medicine (TCM)-related diabetes research, specific TCM prescriptions are administered to address distinct syndromes, such as qi-yin deficiency or yin deficiency with excessive heat.

The pathogenesis of type 2 diabetes is complex, and metabolomic analyses have identified numerous metabolic differences associated with the disease. However, current modeling methods fail to fully replicate the human pathological state of type 2 diabetes. Additionally, inconsistencies in modeling approaches, drug sources, and reagent purity contribute to variability among experimental models. Therefore, the development of novel modeling methodologies remains an urgent challenge in functional metabolomics.

### 3.3 Mechanism verification platform

#### 3.3.1 Molecular simulation platform

Molecular simulation platforms are widely employed for the preliminary screening of drugs and the investigation of sites of action. These technologies can also be utilized for mechanism elucidation or the simulation of cellular processes. Molecular simulations use computational models to predict the interactions between metabolites and biological targets (e.g., proteins, DNA) (Li et al., 2020). For instance, computer simulations can mimic cellular processes or drug-target binding, enabling researchers to prioritize candidates for experimental validation (Qureshi et al., 2020). This approach is cost-effective and expedites hypothesis generation. In one study, simulations elucidated the mechanism by which malaria parasites uptake sugars, guiding subsequent laboratory experiments to confirm these findings. Such tools serve as a critical link between computational predictions and empirical biological insights.

#### 3.3.2 Chemical biology platform

Chemical biology employs labeling techniques (e.g., fluorescent tags, isotopes) to track metabolites in living systems. For example, “click chemistry” allows precise tagging of sugars or proteins without disrupting their function, facilitating the visualization of metabolite distribution and the identification of binding partners. These methods are essential for mapping metabolic pathways and verifying functional roles (Bohara et al., 2024). In 2019, Dong Jiajia et al. from the Shanghai Institute of Organic Chemistry, Chinese Academy of Sciences, unexpectedly discovered a safe and efficient fluorosulfonyl azide (FSO2N3) method (Meng et al., 2019). This method enables azide compounds to undergo cycloaddition reactions with terminal alkyne compounds without requiring separation or purification, allowing for direct functional screening. In 2009, Brett Lomenick et al. (Lomenick et al., 2009) developed a technique known as DARTS (drug affinity-responsive target stability) for target identification. This approach does not require chemical modification of natural products, enabling proteins to retain their native activity and facilitating the identification of direct binding targets of natural products.

#### 3.3.3 Physical biology platform

Beyond cell morphology and number, mechanical properties and microscopic characteristics of cells also exhibit differences in various physiological and pathological states. With advancements in biophysics and nanotechnology, biomechanics has emerged as a new area of exploration (Berthaume and Elton, 2024). Utilizing atomic force microscopy, researchers have demonstrated that cancer cells exhibit reduced stiffness compared to normal cells (Hayashi and Iwata, 2015), which may provide insight into tumor metastasis mechanisms. Bohara et al. employed atomic force microscopy to observe a significant reduction in Young’s modulus, membrane force, membrane tension, and surface adhesion in smooth muscle cells (SMCs) under high glucose conditions, with the lowest values observed in type 2 diabetes mellitus (T2DM)-SMCs (Bohara et al., 2024). Siamantouras et al. (Siamantouras et al., 2016) quantitatively demonstrated the correlation between cell elasticity, adhesion, and early morphological/phenotypic changes in renal tubular injury using atomic force microscopy combined with the Hertz model. Biophysical tools facilitate the study of alterations in cellular physical properties (e.g., stiffness, adhesion) in disease states, providing novel insights into disease progression and therapeutic strategies.

#### 3.3.4 Molecular biology platform

The investigation of the structure, function, and regulatory mechanisms of biological macromolecules such as nucleic acids and proteins is a crucial aspect of functional metabolomics in elucidating disease mechanisms. Various molecular biology techniques are widely applied, continuously optimized, and extensively utilized in the verification phase of omics research. For instance, when anti-inflammatory activity is identified in metabolomics data, cytokines such as IL-10, IL-6, and TGF-β can be detected using specialized kits (Dwivedi and Jena, 2020; Tsuruta et al., 2023), similar to approaches used for other functional metabolites (Perry et al., 2015). Haejin Yoon et al. (Yoon et al., 2020) employed multiple techniques, including immunoprecipitation, western blotting, quantitative RT-PCR, and MT-RNA analysis, to demonstrate that acetyl-CoA carboxylase 2 (ACC2) undergoes hydroxylation and inhibits fatty acid oxidation during high energy expenditure. In PHD3-knockout mice, this hydroxylation process was significantly reduced. The study revealed that AMPK and PHD3 exert opposing effects on fat regulation and exercise capacity. Sharon O. Jensen-Cody et al. (Jensen-Co et al., 2020) demonstrated that FGF21 administration transmits signals to glutamatergic neurons in the ventromedial hypothalamus to suppress sugar intake without increasing energy expenditure. This was elucidated using immunofluorescence, *in situ* hybridization, and single-cell RNA sequencing. Molecular biology techniques (e.g., CRISPR, RNA sequencing) play an essential role in dissecting how metabolites regulate gene and protein function, making them indispensable for linking metabolic alterations to molecular mechanisms.

## 4 Scientific practice of functional metabolomics

### 4.1 Endocrine, nutritional, and metabolic diseases

Zeneng et al. (Wang et al., 2019) conducted a randomized crossover clinical trial involving 113 healthy volunteers. Participants were randomly assigned to three dietary intervention groups for a duration of 4 weeks: red meat, white meat, or no meat. Plasma and urine metabolomics analyses revealed significantly higher levels of TMAO and its precursors in the red meat group compared to the other two groups. Upon cessation of red meat consumption, TMAO levels returned to baseline. Long-term red meat consumption was found to significantly reduce renal TMAO excretion. Isotope tracer studies using d6-choline and d3-carnitine capsules indicated that urinary d6-choline and d6-betaine levels decreased in the white meat group, while choline-derived d6-TMA and d6-TMAO showed no significant differences among groups. These findings suggest that prolonged red meat intake enhances TMAO production via gut microbiota while reducing renal TMAO elimination, potentially increasing the risk of cardiovascular disease, atherosclerosis, and diabetes.

Ruixin Liu et al. (Liu et al., 2017) conducted a metagenome-wide association study on 257 fecal samples obtained from lean and obese young Chinese individuals, confirming the presence of gut dysbiosis in obesity. The study group performed non-targeted metabolomics profiling of serum from patients with type 2 diabetes (T2D) and healthy controls, identifying 148 differential metabolites, including 13 amino acids such as glutamate, phenylalanine, and tyrosine. Targeted metabolomics further validated metabolic disparities between lean and obese individuals. Co-inertia analysis (CIA) and canonical correspondence analysis (CCA) linked these amino acids to alterations in gut microbiota, with glutamate and *Bacteroides* thetaiotaomicron exhibiting a strong association. To investigate the relationship between B. thetaiotaomicron and adiposity, live and heat-killed B. thetaiotaomicron were administered to mice on high-fat and normal diets. Results indicated that B. thetaiotaomicron reduced diet-induced adiposity and influenced serum amino acid levels. Additionally, fecal and serum samples from 23 obese individuals were collected 3 months post-sleeve gastrectomy (SG), revealing that their microbiome and metabolic profiles had become more similar to those of lean controls. This study underscores the association between gut microbiota at the species level, serum amino acids, and adiposity, suggesting potential therapeutic interventions for obesity via gut microbiota modulation.

To identify metabolites associated with T2D, Francois Brial et al. (Brial et al., 2020) employed a targeted GC-MS approach to profile 101 serum metabolites in human subjects. The study identified six metabolites linked to T2D, including 4-cresol, which had not been previously reported in T2D metabolomics research. 4-Cresol exhibited a negative correlation with T2D and may confer protective effects against the disease. Chronic administration of 4-cresol significantly improved glucose homeostasis, enhanced insulin secretion, reduced adiposity, and increased pancreatic mass. Animal studies further demonstrated that 4-cresol treatment promoted β-cell proliferation and pancreatic vascularization, potentially via the downregulation of DYRK1A and upregulation of SIRT1 expression. These findings indicate that 4-cresol, identified through targeted metabolomics, modulates T2D endophenotypes and holds promise for therapeutic applications.

### 4.2 Coronary artery disease

Through a preliminary multi-center, non-targeted clinical metabolomics study of 2,324 clinical serum samples, Lei Zhang et al. (Zhang et al., 2018) identified Neu5AC as the sole sialic acid among 36 differential metabolites in the ESI- ion mode. The research group further identified six metabolites associated with T2D, including 4-cresol, which had not been previously reported in T2D metabolomics studies. Notably, 4-cresol exhibited a negative correlation with T2D and may contribute to disease resistance. Chronic administration of 4-cresol significantly improved glucose homeostasis, enhanced insulin secretion, reduced adiposity, and increased pancreatic weight. Animal studies demonstrated that 4-cresol treatment promoted β-cell proliferation and pancreatic vascularization, potentially through the downregulation of DYRK1A and upregulation of SIRT1 expression. These findings suggest that 4-cresol, identified through targeted metabolomics, plays a regulatory role in T2D endophenotypes and holds potential therapeutic applications.

Zeneng Wang et al. (Wang et al., 2019) conducted a non-targeted metabolomics study on 100 plasma samples from patients with clinical cardiovascular disease and healthy controls. From an initial pool of 18 different small molecules, three metabolites with m/z values of 76, 104, and 118 were selected due to their strong correlation (p = 0.01) with cardiovascular disease. Further analysis identified these metabolites as TMAO, choline, and betaine, all of which exhibited a dose-dependent association with cardiovascular conditions such as peripheral and coronary artery disease in a large clinical cohort (n = 1,876). In murine models, dietary choline and TMAO promoted the enlargement of atherosclerotic plaques, while choline, TMAO, and betaine upregulated the expression of arteriosclerosis-related macrophages. A 3-week broad-spectrum antibiotic pretreatment study confirmed that gut microbiota play an essential role in TMAO production, macrophage cholesterol accumulation, and foam cell formation. Integrative genetic analysis revealed a correlation between hepatic flavin-containing monooxygenase (FMO) gene expression and both TMAO levels and plasma high-density lipoprotein (HDL), potentially elucidating atherosclerosis pathogenesis. These findings establish a mechanistic pathway in which dietary choline or lecithin is metabolized to trimethylamine (TMA) by gut microbiota and subsequently converted to TMAO by hepatic FMO, ultimately contributing to cardiovascular disease progression.

### 4.3 Tumors

Peng Gao et al. (Gao et al., 2016) performed a non-targeted metabolomics analysis of glioma specimens and adjacent control tissues using capillary electrophoresis time-of-flight mass spectrometry (CE-TOF/MS). The study demonstrated significantly elevated levels of hypotaurine in glioma tissues compared to controls. Linear correlation analysis revealed a positive relationship between hypotaurine expression and glioma severity. Molecular docking studies indicated that hypotaurine competes with α-ketoglutarate for binding to prolyl hydroxylase 2, thereby inhibiting its catalytic activity. This inhibition promotes the nuclear translocation of the hypoxia-inducible factor-1α (HIF-1α/β) subunit and upregulates glioma-associated gene expression. Further investigations demonstrated that taurine, the oxidation product of hypotaurine, inhibited intracellular hypotaurine synthesis and consequently suppressed tumor cell proliferation. *In vivo* experiments confirmed that dietary taurine administration slowed tumor growth, suggesting that hypotaurine exerts tumor-promoting effects.

Ling Tang et al. (Tang et al., 2018) analyzed 69 paired liver cancer and adjacent tissue specimens using non-targeted metabolomics based on CE-TOF/MS, followed by quantitative metabolite analysis via gas chromatography-selected ion monitoring mass spectrometry (GC-SIM-MS) and ultra-performance liquid chromatography-multiple reaction monitoring mass spectrometry (UPLC-MRM-MS). Thirteen metabolites were associated with liver cancer, with pathway analysis indicating a positive correlation between hydroxyproline and liver cancer progression. Clinical analysis suggested that low levels of alpha-fetoprotein (AFP) and hydroxyproline might serve as potential biomarkers for liver tumors. *In vitro* experiments demonstrated that exogenous hydroxyproline enhanced cell invasion and increased HIF-1α levels under hypoxic conditions. HIF-1α knockout inhibited cell proliferation. Animal models showed that subcutaneous injection of hydroxyproline-treated SMMC-7221 cells in mice significantly increased tumor volume and HIF-1α expression. Western blot and qRT-PCR analyses confirmed the upregulation of ALDH18A1 and PRODH and the downregulation of PRODH2 under hypoxia, supporting the hypothesis that hydroxyproline promotes liver tumor growth through HIF-1α regulation.

Arun Sreekumar et al. (Sreekumar et al., 2009) identified 1,126 differential metabolites from 262 clinical prostate cancer samples (including 42 tissues, 110 urine, and plasma samples) using high-throughput LC-MS and GC-MS. Metabolic profiling successfully distinguished benign prostate conditions, localized prostate cancer, and metastatic disease. Notably, sarcosine levels were significantly elevated during prostate cancer metastasis, a finding validated in 89 independent samples. Mechanistic studies revealed that sarcosine and its regulatory enzymes (GNMT, SARDH, and DMGDH) may promote tumor progression by enhancing cell invasion and migration. Androgen treatment of ERG-positive and ERG-negative cells, combined with chromatin immunoprecipitation sequencing, linked sarcosine pathway activation to the androgen receptor and ERG genes, which are key mediators in prostate cancer progression. These findings suggest that components of the sarcosine pathway may serve as biomarkers and therapeutic targets for prostate cancer.

### 4.4 Other diseases

Hui Sun et al. (Sun et al., 2018) identified 22 differential metabolic markers in serum samples of Yang Huang syndrome model mice using non-targeted metabolomics. Targeted metabolomics further determined the concentration range of these metabolites, revealing a significant increase in endogenous D-glucuronic acid levels. Subsequent integrative analysis using Ingenuity Pathway Analysis (IPA) identified UDP-glucuronosyltransferase 1A1 as a potential disease target. However, systematic biological regulation experiments on key metabolites and regulatory proteins were not conducted.

Using a mouse model of α-naphthyl isothiocyanate-induced liver injury, Fang Zhongze et al. (Fang et al., 2017) demonstrated significant increases in LysoPC(18:0) and LysoPC(18:1) levels in the serum of the model group compared to healthy controls using UPLC-Q-TOF/MS-based serum metabolomics. Cryopreserved mouse liver tissues were analyzed for mRNA expression of Chka and Scd1, which regulate lipid metabolism. The findings confirmed elevated levels of LysoPC(18:0) and LysoPC(18:1) under pathological conditions. *In vitro* luciferase assays demonstrated that these lysophosphatidylcholines activated NF-κB in a concentration-dependent manner, which was further verified through Western blot and cellular experiments. The use of the NF-κB inhibitor PPARα elucidated the liver injury mechanism involving the NF-κB/IL-6/STAT3 pathway resulting from lipid metabolism dysregulation.

Yanmei Ma et al. (Ma et al., 2015) identified 32 and 37 differential metabolites in tilapia infected with *Streptococcus* evansi (LD50 lethal group) and the survival group, respectively, using non-targeted GC-MS metabolomics at 30°C. Pathway analysis revealed L-leucine as the most significant metabolite distinguishing the LD50 and survival groups. To validate its protective effects against *Streptococcus* iniae infection, the authors administered exogenous L-leucine via injection and oral supplementation. Both methods significantly improved survival rates in a dose-dependent manner, indicating that L-leucine enhances resistance to bacterial infections.

Pandey et al. (Pa and ndey, 2024) systematically analyzed the plasma metabolic profiles of patients with sepsis and septic shock using metabolomics technologies, identifying key metabolites such as tryptophan degradation products and acylcarnitines associated with inflammatory storms and mitochondrial dysfunction. Dynamic monitoring of succinate and lactate accumulation further demonstrated their strong correlation with the risk of multi-organ failure in septic shock, providing real-time prognostic markers for clinical stratification (Pandey, 2024). These findings provide potential biomarkers and novel therapeutic strategies for early diagnosis, risk stratification, and targeted metabolic interventions. Additionally, another study utilized metabolomics to analyze the serum metabolic profiles of patients with acute respiratory distress syndrome (ARDS) complicated by acute kidney injury (AKI), revealing metabolic pathway abnormalities associated with inflammation and oxidative stress (Singh et al., 2024). Furthermore, extensive applications in critical care have demonstrated that mitochondrial dysfunction can be systematically elucidated via TCA cycle metabolite flux analysis, providing mechanistic insights into the collapse of energy metabolism during multi-organ failure (Siddiqui et al., 2020). These insights provide a foundation for understanding multiple organ failure (MOF) pathogenesis and developing targeted metabolic therapies.

Currently, studies focusing on functional metabolomics remain limited. Table 2 summarizes key metabolites identified in the aforementioned studies.

**TABLE 2:** Information about key metabolites of functional metabolomics based on pathological conditions.

No	Metabolite	Formula	Molecular weight	Precursor method*	Level	Sample category	Functional verification	Validation model	Disease	Ref
1	D-Glucuronic acid	C6H10O7	194.1	C	↑	Mice/Serum	×	Not validated experimentally	Yang Huang syndrome	Sun et al. (2018)
2	LysoPC(18:0)	C26H54NO7P	523.7	A	↑	Mice/Serum	√	LO2 cells: Human hepatocyte model for liver injury studies	Cholestatic liver injury	Fang et al. (2017)
3	LysoPC(18:1)	C26H52NO7P	521.7	A	↑	Mice/Serum	√	LO2 cells: Human hepatocyte model for liver injury studies	Cholestatic liver injury	Fang et al. (2017)
4	Neu5AC	C11H19NO9	309.3	C	↑	Human/Serum	√	SD rats: sprague-Dawley rats for myocardial injury modeling Ventricular myocytes model	Coronary Artery Diseases	Zhang et al. (2018)
5	5-Methoxytryptophan	C12H14N2O3	234.3	C	↓	Human/Serum	√	HK-2 cells: Human renal proximal tubule cells HMC cells: Human mesangial cells Mice: CKD model	Chronic kidney disease	Chen et al. (2019)
6	Hydroxyproline	C5H9NO3	131.1	C	↑	Human/Tissue	√	SMMC-7721/HepG2 cells: Liver cancer cell lines Mice: Xenograft tumor model	Hepatocellular carcinoma	Tang et al. (2018)
7	Hypotaurine	C2H7NO2S	109.2	C	↑	Human/Tissue	√	U87/U251 cells: Glioblastoma cell lines Nude mice: Subcutaneous tumor model	Gliomas	Gao et al. (2016)
8	4-Cresol	C7H8O	108.1	B	↓	Human/Serum	√	C57BL/6J mice: Type 2 diabetes model Islet cells: *In vitro*insulin secretion assay	Type II diabetes	Brial et al. (2020)
9	Phenyl sulfate	C6H5O4S	173.2	C	↑	Mice/Serum	√	db/db mice: Leptin receptor-deficient diabetic model Rat: Diabetic nephropathy model	Diabetic nephropathy	Kikuchi et al. (2019)
10	L-leucine	C6H13NO	131.2	A	↑	Liver/Tilapias	√	Tilapias: Bacterial infection model Mice: Immunocompetent host validation	*Streptococcus* infection	Ma et al. (2015)
11	Imidazole Propionate	C6H8N2O2	140.14	C	↑	Human/Plasma Mice/Plasma	√	Hepatocyte: Primary liver cells Mice: type 2 diabetes models	Type II diabetes	Koh et al. (2018)
12	Trimethylamine oxide	C3H9NO	75.11	B	↑	Human/Plasma Human/Urine	×	-	Nutritional intervention	Wang et al. (2019)
13	L-carnitine	C7H15NO3	161.20	B	↑	Human/Plasma Human/Urine Human/fecal	√	Apoe^−/−^ mice: Atherosclerosis model	Cardiovascular disease	Koeth et al. (2013)
14	Choline	C5H14NO+	104.17	C	↑	Human/Plasma	√	Apoe^−/−^ mice: Atherosclerosis model; Human cohorts: Clinical biomarker analysis	Cardiovascular disease	Wang et al. (2011)
15	Betaine	C5H11NO2	117.15	C	↑	Human/Plasma	×	Apoe^−/−^ mice: Atherosclerosis model (mechanism inferred from indirect evidence)	Cardiovascular disease	Wang et al. (2011)
16	L-Glutamic acid	C5H9NO4	147.13	C	↑	Human/Serum	√	*Bacteroides* thetaiotaomicron: Gut microbiota modulation in obesity model	obesity	Liu et al. (2017)
17	Sarcosine	C3H7NO2	89.09	C	↑	Human/Plasma Human/Urine Human/Tissue	×	DU145/VCaP/LNCaP cells: Prostate cancer cell lines; Clinical cohorts: Metastasis correlation	Prostate cancer	Sreekumar et al. (2009)

Note: A:Non targeted metabolomics; B: targeted metabolomics; C:Non-targeted + targeted metabolomic.

## 5 Limitations

As a frontier field within metabolomics, functional metabolomics offers unique advantages in elucidating the biological functions of metabolites; however, it still faces several limitations. The primary limitation is the incompleteness of metabolite databases. Currently, public databases such as HMDB, METLIN, and LipidMaps encompass only a fraction of the metabolites present in actual biological samples, particularly plant secondary metabolites, microbial metabolites, and novel modified metabolites such as acylated amino acids (Wishart et al., 2022). This limitation results in a substantial number of unannotated metabolites, thereby impeding the in-depth analysis of their functional mechanisms. Furthermore, standardization in experimental procedures remains insufficient. Variability in sample preparation and data analysis across different laboratories compromises the comparability of research findings (Blaženović et al., 2019). Additionally, technical challenges persist in functional metabolomics. While mass spectrometry and NMR spectroscopy are widely utilized for metabolite detection, both techniques have inherent limitations in sensitivity and accuracy. Although mass spectrometry exhibits high sensitivity, it encounters difficulties in metabolite identification within complex biological matrices, whereas NMR spectroscopy is constrained by both sensitivity and resolution. To advance functional metabolomics, it is imperative to address these limitations by developing more comprehensive metabolite databases, establishing standardized experimental protocols, and enhancing detection technologies to improve metabolite identification accuracy and facilitate in-depth functional investigations.

## 6 Conclusion and prospect

While traditional metabolomics has identified numerous phenotype-associated biomarkers, the biological relevance and functional mechanisms of these metabolites remain inadequately explored. Functional metabolomics has emerged as a critical paradigm shift, transitioning from metabolite identification to mechanistic validation through advanced analytical platforms and isotope labeling techniques. Future advancements should focus on four strategic directions (Oliverp et al., 1998): Multi-omics integration, incorporating genomics, proteomics, and lipidomics to establish causal relationships between metabolites and disease pathways (Nicholson et al., 1999); Application of emerging technologies, including single-cell metabolomics, spatial metabolomics, and AI-driven metabolic network modeling, to resolve tissue-specific metabolic heterogeneity (Fiehn et al., 2000); Personalized medicine initiatives, aimed at identifying individual-specific metabolic profiles and biomarkers to facilitate the development of more effective treatment strategies in clinical practice; and (Mayerle et al., 2018) Clinical translation, utilizing functional metabolite panels for drug target discovery and personalized therapeutic interventions. Notably, the convergence of stable isotope tracing with CRISPR screening platforms holds the potential to systematically map metabolite-protein interactions. This evolution from descriptive biomarker discovery to mechanism-driven research will accelerate precision medicine, particularly in fields such as neurodegenerative diseases and cancer metabolism.

Additionally, functional metabolomics has experienced substantial advancements in recent years, largely driven by the integration of chemistry, biology, and computational science. To further propel the field, it is essential to foster interdisciplinary research by promoting collaboration among experimentalists, clinicians, and data scientists. We advocate for the establishment of interdisciplinary alliances to facilitate shared platforms for standardized data exchange and joint training programs aimed at cultivating hybrid expertise. For example, integrating chemical isotope labeling with machine learning techniques can significantly enhance metabolite annotation accuracy, while systems biology approaches can effectively link metabolic pathways to disease phenotypes. This synergy will not only drive innovation in biomarker discovery but also advance therapeutic targeting strategies.
